# Peritoneal flaps for the prevention of lymphoceles after robot-assisted radical prostatectomy-a systematic review and IPD-meta-analysis of randomized controlled trials

**DOI:** 10.1007/s00345-026-06254-y

**Published:** 2026-02-24

**Authors:** Gloria Baumann, Caelán Max Haney-Aubert, Victoria Luise Simone Wieland, Jiri Lehmberg, Wahid Fattal, Christian Gilfrich, Maximilian Burger, Vladimir Student, Joseph Wagner, Simon Gloger, Maurice Stephan Michel, Karl-Friedrich Kowalewski, Manuel Neuberger

**Affiliations:** 1 Medical Faculty Mannheim, Department of Urology and Urosurgery, University Medical Center Mannheim, Heidelberg University, Theodor-Kutzer-Ufer 1-3, 68167 Mannheim, Germany; 2 German Cancer Research Center (DKFZ) Heidelberg, Division of Intelligent Systems and Robotics in Urology (ISRU), Heidelberg, Germany; 3 DKFZ Hector Cancer Institute at the University Medical Center Mannheim, Mannheim, Germany; 4 Department of Uroloogy, St. Elisabeth Hospital Straubing, Straubing, Germany; 5 Department of Urology, University of Regensburg, Caritas St. Josef Medical Center, Regensburg, Germany; 6 Department of Urology, University Hospital Olomouc, Olomouc, Czech Republic; 7 Department of Urology, Hartford Healthcare Medical Group, Hartford Hospital, Hartford, CT USA; 8 Center for Minimally Invasive and Robotic Urology, Augusta Hospital Bochum, Witten/Herdecke University, Bochum, Germany

**Keywords:** Pelvic lymph node dissection (PLND), Surgical technique, Randomized controlled trials, Evidence-based surgery, Meta-analysis, Robot-assisted radical prostatectomy (RARP), Lymphocele

## Abstract

**Purpose:**

Lymphoceles (LCs) are one of the most common complications after robot-assisted radical prostatectomy (RARP). Peritoneal flaps (PF) have shown to possibly decrease the incidence of LCs. This individual patient data meta-analysis (IPD-MA) investigates the efficacy of PF on rates of symptomatic lymphoceles (sLCs) requiring intervention, sLCs, overall lymphoceles and perioperative complications.

**Methods:**

We conducted a prespecified systematic review and IPD-MA (PROSPERO CRD42024519284). A database search via Medline, Web of Science, Embase, Central, ClinicalTrials.gov and the International Clinical Trials Registry Platform was performed up to December 2023. Six randomized controlled trials (RCTs) comparing PF vs. no PF during RARP with PLND were included and five RCTs provided IPD. MA was conducted using a random-effects model (two-stage model).

**Results:**

PF significantly reduced sLCs requiring intervention as well as the rates of sLCs and overall lymphoceles. Additionally, PF significantly reduced overall complications, most likely driven by fewer lymphoceles and associated interventions. Furthermore, BMI and the presence of lymphoceles at discharge were identified as risk factors for sLCs. Subgroup analyses suggested that the protective effect of PF diminishes with increasing BMI, indicating limited benefit in patients with severe obesity.

**Conclusions:**

The creation of PF after RARP significantly reduces sLCs without affecting the complication rate or meaningfully prolonging operative time. LCs at the time of discharge represent significant risk factors for occurrence of sLCs, indicating a possibility for closer observation for this subgroup. With the evidence provided by this IPD-MA, creation of PF should be the new standard of care to reduce LC-related morbidity.

**Supplementary Information:**

The online version contains supplementary material available at 10.1007/s00345-026-06254-y.

##  Background

Prostate cancer (PC) is the second most common cancer worldwide and a surge in cases is expected in the near future [1, 2]. When treated surgically, robot-assisted radical prostatectomy (RARP) has become the standard approach and has shown to provide superior short-term continence and long-term potency compared to conventional laparoscopic radical prostatectomy [3]. Pelvic lymph node dissection (PLND) is a standard procedure for operative lymph node staging during RARP, and recent studies have suggested an added oncologic benefit [4]. One of the most relevant postoperative complications is the development of lymphoceles (LC). Symptomatic LCs (sLC), i.e. fluid formation near the LND area in combination with lower abdominal pain, infection, leg swelling or thrombosis, require intervention and can be treated with either drain insertion (with or without instillation of sclerosing agents) or surgical fenestration. Infected lymphoceles, in particular, are associated with significant morbidity. Not only sLCs but also asymptomatic lymphoceles (aLCs) may become clinically relevant over time. Given their anatomical proximity to the iliac vessels, aLCs may lead to venous compression and carry a potential risk of secondary complications such as deep venous thrombosis or progression to symptomatic disease. Therefore, both sLCs and aLCs contribute to a relevant clinical burden for patients and the health-care system.

In recent years, several randomized controlled trials (RCT) have investigated the benefit of peritoneal flaps (PF) during RARP with PLND to reduce postoperative LCs [5–10].

This systematic review and meta-analysis (MA) aims to analyze the impact of PF on postoperative sLC requiring intervention as the primary endpoint and formation of all LCs and postoperative complications based on individual patient data (IPD).

##  Methods

This systematic review and IPD-MA was performed in line with the Preferred Reporting Items for Systematic Reviews and Meta-Analyses statement and prospectively registered via PROSPERO (CRD42024519284). The study was conducted according to a prospectively developed protocol.

### Eligibility criteria, search, data acquisition

A database search via Medline, Web of Science, Embase, Central, ClinicalTrials.gov and the International Clinical Trials Registry Platform was performed by an experienced librarian using key words, MeSH terms and Boolean connectors. The last date of the search was 18.12.2023. There were no restrictions regarding language. The PICOS were predefined and listed in the trial registration.

- **P**: Patients with prostate cancer undergoing radical prostatectomy with pelvic lymph node dissection
- **I**: Peritoneal flap after radical prostatectomy with pelvic lymph node dissection
- **C**: No peritoneal flap after radical prostatectomy with lymph node dissection
- **O**: Lymphoceles requiring intervention (primary outcome), any secondary outcome
- **S**: Only randomized controlled trials

Subsequently, title and abstract screening of the references were performed by two trial authors (CMH, VW). Full texts were acquired where possible and reviewed for inclusion by the same two authors. After the trials were identified, the trial teams were contacted for IPD via email, telephone and personal contacts. IPD for one trial (PELYCAN) was already available.

Data collection was performed using a predefined excel data sheet. Authors of the trials either transposed the data themselves or provided the trial data and it was transposed by the systematic review team. Uncertainties were resolved by contacting the trial authors.

### Primary outcome

The primary outcome was the occurrence of sLCs requiring intervention. Intervention included insertion of a drain (with or without instillation of a sclerosing agent) and surgical fenestration of the lymphocele. A lymphocele was defined as a fluid collection adjacent to the area of lymph node dissection.

### Secondary outcomes

Secondary outcomes included symptomatic lymphoceles, lymphocele status (i.e. asymptomatic or symptomatic lymphoceles) and complications.

### Risk of bias and certainty of evidence

Risk of bias was assessed with the risk of bias 2.0 tool and certainty of evidence was assessed with the GRADE tool.

### Subgroup analyses

Subgroup analyses for the primary outcome were performed by age (< 65 vs. ≥ 65 years), PSA value (< 5, 5–10, > 10 ng/mL), BMI (< 30 vs. ≥ 30 kg/m²), and by the number of resected lymph nodes, used as a pragmatic surrogate for the extent of pelvic lymph node dissection (extended vs. limited PLND).

### Risk factors of symptomatic lymphoceles

In addition to the stated outcomes, a prediction model for sLCs requiring intervention was developed as multilevel logistic regression. The following variables were used within this model: age, body mass index, PSA value, number of lymph nodes resected, lymphocele visibility at discharge and peritoneal flap performed.

### Statistical analysis

Analyses were performed using IPD where available and aggregate data for studies that did not supply IPD. Unless otherwise stated, effect estimates are presented as odds ratios (ORs) with 95% confidence intervals (CI). All tests were two-sided and a p-value < 0.05 was considered statistically significant. A one-stage mixed-effects logistic regression model was fitted to the IPD, with a random intercept for study to account for between-trial heterogeneity. The model included treatment group (peritoneal flap vs. no flap), age, body mass index (BMI), and preoperative PSA. Continuous covariates were standardized to improve model convergence. To combine the IPD treatment effect with the aggregate trial results, a two-stage random-effects meta-analysis was performed using the IPD mixed model results and aggregate data. For a complementary conventional meta-analysis, we reconstructed 2 × 2 tables from the IPD for each trial and combined these with the Pose data and random-effects model was fitted using REML and the log-OR as the effect measure. Between-study heterogeneity was summarised using τ² and I² statistics. Conventional forest plots display study-specific and pooled ORs on a log scale. Funnel plots were generated for the primary outcome to visually assess small-study effects. The consistency of the flap effect across clinically relevant subgroups was examined for the primary outcome. Predefined subgroups were: age < 65 vs. ≥ 65 years; PSA < 5, 5–10, and > 10 ng/mL; BMI < 30 vs. ≥ 30 kg/m²; lymph node count < 15 vs. ≥ 15; and PLND type (limited vs. extended). For each subgroup, we fitted logistic regression models including treatment and relevant adjustment covariates, omitting the variable used for stratification from the adjustment set. Within each subgroup level we estimated the adjusted OR for flap vs. no flap. To formally test for effect modification, we added an interaction term between treatment and the subgroup variable (treatment × subgroup) in a single model and used likelihood ratio (or Wald) tests (via drop1) to obtain P-values for interaction. Results are summarized in subgroup tables and visualised in a forest plot. To explore predictors of symptomatic lymphocele, we fitted a multivariable logistic regression model to the IPD which included treatment group, age, BMI, PSA, number of resected lymph nodes, and lymphocele at discharge (yes/no) as a postoperative predictor, restricting the analysis to studies and patients with complete data on this variable.

All analyses were performed in R (R Foundation for Statistical Computing). Mixed-effects models were fitted using the lme4 package (glmer), meta-analyses using the metafor package (rma), data wrangling with dplyr and tidyr, and visualisations (including forest and funnel plots) with forestplot and base R graphics.

##  Results

### Study characteristics

**Fig. 1:**
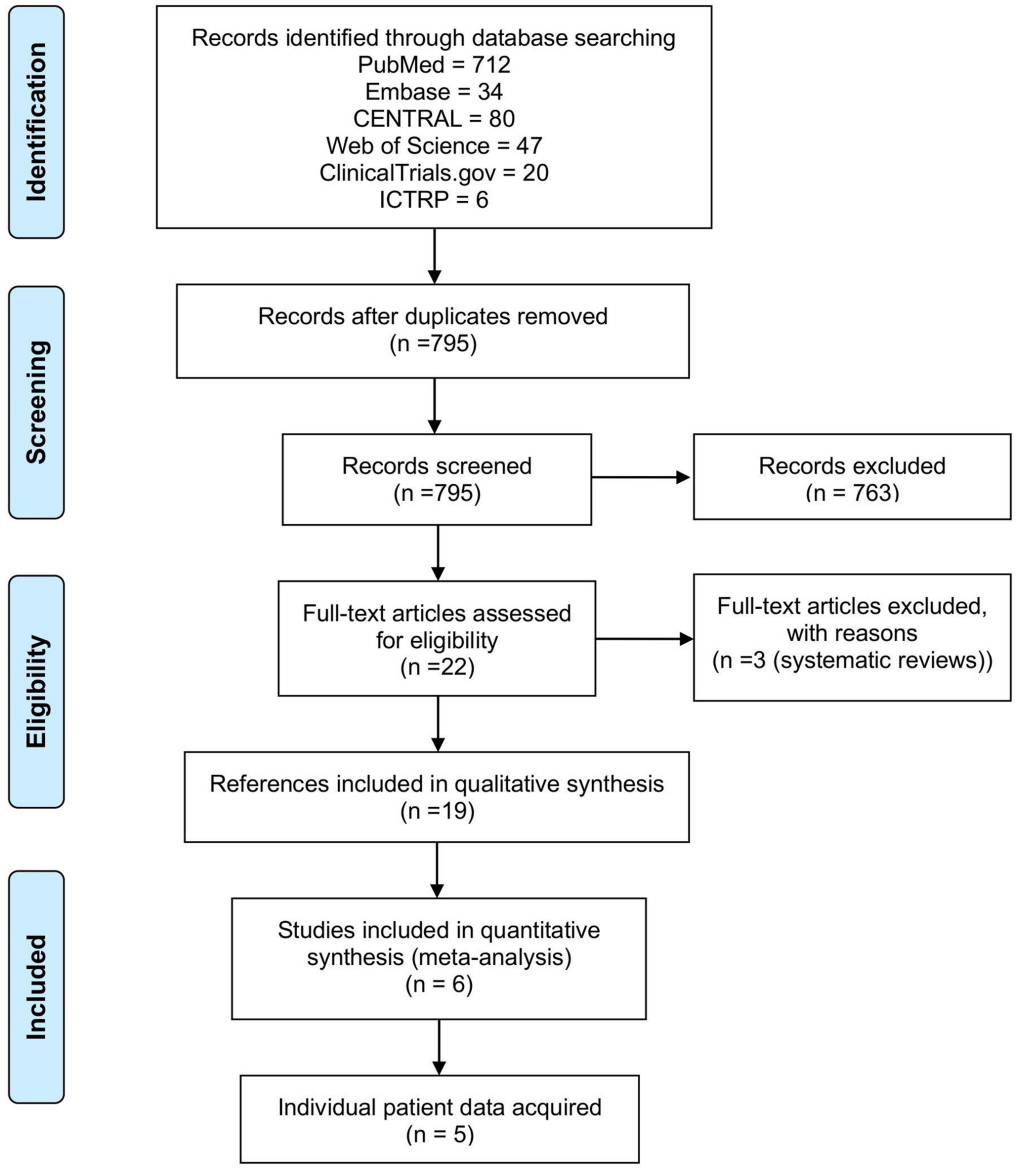
PRIMSA flow diagram of the study selection process

After removal of duplicates, the search identified 795 records. Six individual RCTs were identified. After contact with the trial authors, five trials provided IPD, and for one trial summary data extracted from the trial report and supplementary material was available. A detailed overview of the study selection process is provided in the PRISMA flow diagram (Fig. 1). An overview of the included studies is available in the supplementary material (Supplementary Table S1). Baseline variables were generally comparable across trials; two studies were multicentric and four were single-center. All studies evaluated some form of lymphocele as their primary outcome. A detailed summary of baseline patient characteristics is provided in Table 1. Information about operative and methodological aspects of each trial, including the PF technique, follow-up schedules, blinding, and PLND templates, is summarized in the supplementary material (Supplementary Table S2).

**Table 1:** Baseline patient characteristics for experimental groups (Flap and no Flap)

Age [years] Median (IQR)		PROLY (2022)	PERFIX (2023)	PIANOFORTE (2020)	PLUS (2023)	PELYCAN (2023)	POSE TRIAL (2025)
	FLAP	65 (60–70)	66 (47–76)	64.5 (59.5–69)	63 (59–68)	68.5 (63.7–72.3)	Not reported
	NO FLAP	66 (60–70)	66 (46–80)	66 (60.5–66)	64 (59–68)	67.2 (61.6–71.4)	Not reported
BMI [kg/m²] Median (IQR)							
	FLAP	26 (25–29)	28.1 (19.72–43.28)	27.4 (25.6–29.7)	28.8 (26.9–32)	26.8 (24.2–30.5)	Not reported
	NO FLAP	27 (25–30)	28.9 (20.57–43.94)	27.2 (25–29.9)	27.8 (25.7–32.3)	26.9 (24.8–29.4)	Not reported
ASA (≥ II) *n* (%)							
	FLAP	209 (89.0)	Not reported	Not reported	Not reported	224 (82.96)	Not reported
	NO FLAP	194 (84.0)	Not reported	Not reported	Not reported	241 (87.96)	Not reported
PSA [ng/mL] Median (IQR)							
	FLAP	6.9 (5.3–9.9)	7.2 (0.6–56)	8.1 (6.2–13.5)	6.8 (5.3–9.7)	7.1 (5.4–10.8)	Not reported
	NO FLAP	7.4 (5.2–12.0)	7.6 (1.7–93)	8.3 (6.0–12.9)	6.5 (4.9–9.5)	7.4 (5.5–10.8)	Not reported
Prostatic volume (11) Median (IQR)							
	FLAP	39 (30–55)	60^1^ (30–157)	Not reported	Not reported	26.8 (24.2–30.5)	Not reported
	NO FLAP	40 (30–53)	60.5^1^ (25–150)	Not reported	Not reported	26.9 (24.8–29.4)	Not reported
High risk PC (preoperative) *n* (%)							
	FLAP	Not reported	58^2^ (47.2)	Not reported	31^3^ (28.2)	73^4^ (27.0)	Not reported
	NO FLAP	Not reported	61^2^ (50.0)	Not reported	25^3^ (23.6)	74^4^ (27.0)	Not reported
Pathological ISUP (≥ 3) *n* (%)							
	FLAP	101 (42.26)	78 (63.41)	46 (42.59)	Not reported	113 (41.85)	Not reported
	NO FLAP	93 (39.41)	72 (59.01)	49 (39.52)	Not reported	101 (36.86)	Not reported
Pathological tumor stage (≥ pT3) *n* (%)							
	FLAP	109 (45.61)	72 (58.54)	36 (33.33)	38 (34.55)	85 (31.48)	Not reported
	NO FLAP	82 (34.75)	72 (59.01)	31 (25.0)	49 (46.23)	95 (34.67)	Not reported
Lymph node count Median (IQR)							
	FLAP	14 (11–18)	17 (6–19)	15 (10–22)	15 (11–19)	12 (8–16)	14 (not reported)
	NO FLAP	14 (11–19)	17 (7–56)	16 (11–21)	16 (12–21)	11 (8–16)	14 (not reported)

Baseline characteristics were extracted from original publications or derived from individual patient data where not explicitly reported, (*ASA* American Society of Anesthesiologists Physical Status classification, *BMI*  Body mass index, *IQR* interquartile range, *PC* prostate cancer, *PSA* prostate specific antigen

^1^For the PerFix trial, prostatic volume was reported as prostate weight (g) and is presented in milliliters assuming g ≈ mL

^2^According to EAU risk group (European Association of Urology)

^3^According to NCCN risk group (National Comprehensive Cancer Network); ^4^ According to D’Amico risk classification)

### Primary outcome

**Fig. 2:**
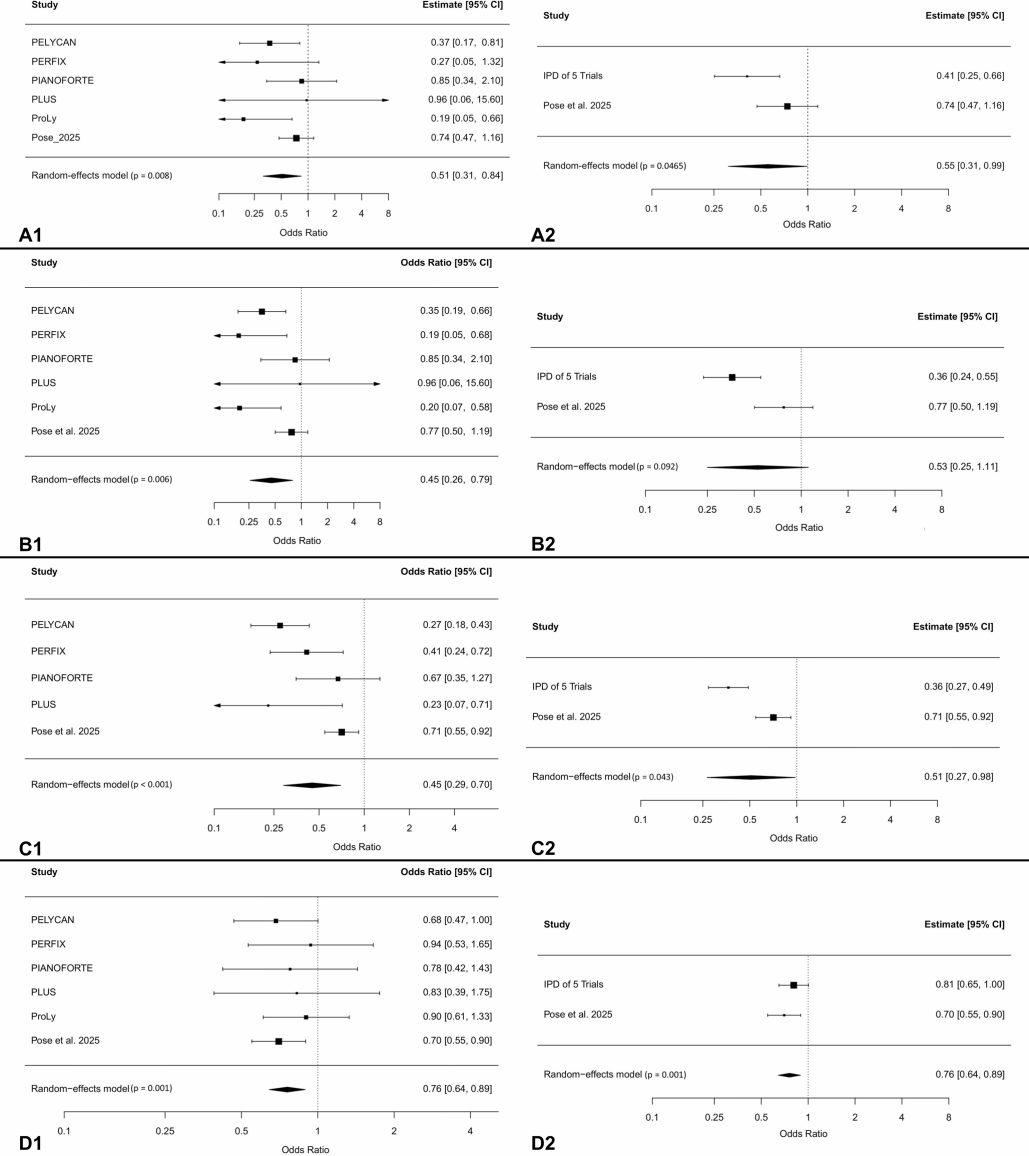
Forest Plots displaying Results from Conventional and IPD Meta-Analysis for the Primary and Secondary Outcomes: Odds ratios (OR) and 95% confidence intervals (CI) for each outcome comparing peritoneal flap (PF) vs. no PF. **Panel A**: symptomatic lymphoceles (sLCs) requiring intervention (= primary outcome); **Panel B**: symptomatic lymphoceles (SLCs); **Panel C**: overall lymphoceles; **Panel D**: overall complications. **Left (A1 – D1)**: conventional meta-analyses; **Right (A2 – D2)**: IPD-only analyses. Horizontal lines represent 95% confidence intervals; the vertical line marks no effect (OR = 1)

Data on symptomatic lymphoceles requiring intervention were available for all trials. Absolute and relative frequencies for each trial and treatment group are summarized in Table 2. With high certainty of evidence, there was a statistically significant reduction associated with peritoneal flaps (OR 0.51; 95% CI 0.31–0.84; *p* = 0.008). This result did not vary substantially when only analyzing trials that provided IPD (OR 0.41; 95% CI 0.25–0.66; *p* < 0.001). These findings are illustrated in Fig. 2A (A1 + A2), showing a consistent reduction with PF across both analyses (conventional MA and IPD-MA). As only one trial provided time-to-event data on interventions and last follow-up, no time-to-event analysis was performed.

**Table 2:** Incidence of lymphoceles during Follow-up in included randomized controlled trials for experimental groups (Flap vs. No Flap)

Symptomatic LCs requiring intervention *n* (%)		PROLY (2022)	PERFIX (2023)	PIANOFORTE (2020)	PLUS (2023)	PELYCAN (2023)	POSE TRIAL (2025)
	FLAP	3 (1.3)	2 (7.4)	9 (8.3)	1 (0.9)	10 (3.7)	38 (7.2)
	NO FLAP	16 (6.8)	7 (14.0)	12 (9.7)	1 (0.9)	25 (9.1)	48 (8.8)
Symptomatic LCs *n* (%)							
	FLAP	8 (3.3)	3 (2.4)	9 (8.3)	1 (0.9)	10 (3.7)	Not reported
	NO FLAP	19 (8.1)	14 (11.5)	12 (9.7)	1 (0.9)	25 (9.1)	Not reported
Overall LCs *n* (%)							
	FLAP	52 (22.0)	27 (22.0)	19 (17.6)	4 (3.6)	34 (12.3)	Not reported
	NO FLAP	77 (33.0)	50 (41.0)	30 (24.2)	15 (14.2)	94 (34.3)	Not reported

Exact follow-up durations and assessment time points differed between trials and are detailed in the Supplementary Material (*LCs* lymphoceles)

### Secondary outcomes

#### Further lymphocele outcomes

Absolute and relative frequencies of symptomatic lymphoceles and overall lymphoceles stratified by trial and treatment group are provided in Table 2. With high certainty of evidence, the conventional MA and the IPD analyses both demonstrated a statistical significant reduction in sLCs (conventional analysis: OR 0.45; 95% CI 0.26–0.79; *p* = 0.006; IPD-only analysis: OR 0.36; 95% CI 0.24–0.55; *p* < 0.001).

Also for overall lymphoceles, both analyses demonstrated statistically significant reductions (conventional analysis: OR 0.45; 95% CI 0.29–0.70; *p* < 0.001; IPD-only analysis: OR 0.36; 95% CI 0.27–0.49; *p* < 0.001). The corresponding forest plots for sLCs and overall LCs are shown in Fig. 2B (B1 + B2) and Fig. 2C (C1 + C2), respectively.

#### Complications

In the conventional MA, a statistically significant reduction in overall complications was observed in patients receiving PF with moderate certainty of evidence (OR 0.76; 95% CI 0.64–0.89; *p* = 0.001). In contrast, when restricting the analysis to the five IPD studies, this effect just missed statistical significance (OR 0.81; 95% CI 0.65–1.00; *p* = 0.055). When stratifying complication severity, the conventional MA demonstrated a statistically significant reduction in minor complications (OR 0.77; 95% CI 0.65–0.92; *p* = 0.004), while no significant effect was observed for major complications (OR 0.89; 95% CI 0.68–1.16; *p* = 0.373). In the IPD-only analyses, neither minor nor major complications reached statistical significance. The forest plots for overall complications are presented in Fig. 2D (D1 + D2).

#### Operative time, postoperative hospital stay, blood loss

There were no statistically significant differences for all three outcomes in the IPD-MA. Operative time showed a pooled mean difference of 1.74 min (95% CI 4.86–8.34; *p* = 0.61). Postoperative hospital stay differed by a mean difference of 0.20 days (95% CI 0.10–0.50; *p* = 0.19). Estimated blood loss had a pooled mean difference of 8.65 mL (95% CI 9.48–26.78; *p* = 0.35).

### Risk of bias and certainty of evidence

Certainty of evidence as assessed via GRADE was rated as high for sLCs requiring intervention, sLCs and overall LCs (Table 3). The certainty of evidence regarding overall complications was rated as moderate due to imprecision and selective reporting.

**Table 3:** Summary of findings and GRADE assessment for the Meta-Analysis

Outcomes	Anticipated absolute effects* (95% CI)		Relative effect (95% CI)	№ of participants (studies)	Certainty of the evidence (GRADE)
	Risk with standard of care	Risk with Peritoneal flaps			
Symptomatic lymphoceles requiring intervention	85 per 1.000	**45 per 1.000** (27 to 74)	OR 0.51 (0.30 to 0.87)	2797 (6 RCTs)	⊕⊕⊕⊕ High
Symptomatic lymphoceles	97 per 1.000	**50 per 1.000** (37 to 66)	OR 0.49 (0.36 to 0.66)	2797 (6 RCTs)	⊕⊕⊕⊕ High
Lymphocele status (i.e. symptomatic lymphocele or asymptomatic lymphocele)	188 per 1.000	**90 per 1.000** (63 to 131)	OR 0.43 (0.29 to 0.65)	1717 (5 RCTs)	⊕⊕⊕⊕ High
Overall complications	249 per 1.000	**218 per 1.000** (184 to 257)	OR 0.84 (0.68 to 1.04)	1717 (5 RCTs)	⊕⊕⊕⊕◯ Moderate

*The risk in the intervention group (and its 95% confidence interval) is based on the assumed risk in the comparison group and the **relative effect** of the intervention (and its 95% CI)

*CI*: confidence interva, *OR* odds ratio, *RCTs* randomized controlled trials

**GRADE Working Group grades of evidence**

**High certainty**: we are very confident that the true effect lies close to that of the estimate of the effect

**Moderate certainty**: we are moderately confident in the effect estimate: the true effect is likely to be close to the estimate of the effect, but there is a possibility that it is substantially different

**Low certainty**: our confidence in the effect estimate is limited: the true effect may be substantially different from the estimate of the effect

**Very low certainty**: we have very little confidence in the effect estimate: the true effect is likely to be substantially different from the estimate of effect

Overall, the risk of bias was low to moderate. In four of five IPD trials, the patients were blinded to their assignment, and in 3/6 studies the randomization was performed intraoperatively, thereby minimizing surgeon bias [6, 7, 10]. Postoperative teams were blinded in 2/6 trials [7, 8] and the outcome assessors in 4/6 trials [5–7, 10]. The primary outcome was objective and therefore at low risk of bias. The complete risk of bias assessment is presented in a supplementary traffic-light plot (Figure S1). As an exception, Pose et al. [9] was rated as high risk due to incomplete data.

### Subgroup analyses

In our subgroup analyses, the PF reduced the risk of sLCs requiring intervention across strata. The treatment effect did not differ by age (p_interaction_ = 0.959) and showed no heterogeneity by PSA (p_interaction_ = 0.894). In contrast, there was clear effect modification by BMI: patients without obesity (BMI < 30 kg/m²) benefited significantly, whereas those with obesity (BMI ≥ 30 kg/m²) did not (p_interaction_ = 0.003). Results were consistent across lymph node yield (< 15 vs. ≥ 15 resected nodes), with no evidence of heterogeneity (p_interaction_ = 0.678). The complete results of the subgroup analyses are presented in Table 4.

**Table 4:**
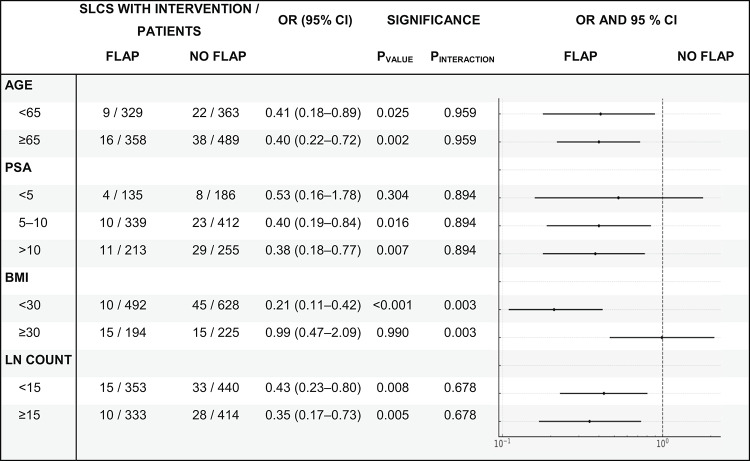
Subgroup Analyses for the Primary Outcome (sLCs requiring intervention): odds ratios of peritoneal flap vs. no flap within each subgroup and corresponding Wald *p*-values; interaction *p*-values test for heterogeneity of the treatment effect across subgroup levels

*BMI* body mass index, *CI* confidence interval, *LN* lymph node, *OR* odds ratio, *PSA* prostate-specific antigen

### Risk factors of symptomatic lymphoceles

In addition to the stated outcomes, a prediction model for sLCs requiring intervention was developed. In this multilevel logistic regression model, higher BMI was a significant risk factor (OR = 1.06; 95% CI 1.01–1.11; *p* = 0.01), and visible lymphoceles at discharge showed the strongest association with sLCs (OR = 3.38; 95% CI 2.08–5.38; *p* < 0.001). The use of a peritoneal flap was significantly protective (OR = 0.39; 95% CI 0.24–0.62; *p* < 0.001). The fixed-effects estimates for all predictors included in this model are provided in the supplementary material (Supplementary Table S3).

##  Discussion

This MA comprises results of all six available RCTs regarding prevention of LCs by creating PFs following RARP with PLND. Furthermore, it is the first one to contain analyses based on IPD for which five of the six trials provided data. It was demonstrated with high certainty of evidence that the creation of PFs during RARP reduced formation of sLCs requiring intervention as well as aLCs significantly. In the analyses based on IPD the risk for sLC was reduced by 45% (OR 0.55); the conventional MA of all published results showed a 49% reduction (OR 0.51). These results are comparable to previous MAs of RCTs which reported an OR of 0.35 to 0.46 [11–15].

A particularly relevant finding of this MA is the strong protective effect of PFs against early aLC formation: across all follow-up periods, PFs reduced the rate of aLC by 46%. Regarding aLCs at the time of discharge, three of the six studies examined the impact of PFs on aLCs at the time of discharge. Here, the PELCYAN study found a significant reduction of 54% [6]. Notably, early aLC at discharge emerged as a strong predictor for later sLCs requiring intervention. While routine screening for lymphoceles prior to discharge is currently not recommended by guidelines and therefore not yet standard clinical practice, our findings suggest that the presence of early aLC identifies a subgroup of patients at particularly high risk for subsequent clinically relevant lymphoceles. Based on the evidence provided by this IPD-MA, selective postoperative screening strategies (e.g., targeted ultrasonography) in patients undergoing RARP with PLND may be considered to identify this vulnerable subgroup with early aLCs, that could benefit from closer postoperative follow-up. This may facilitate earlier detection of complications in outpatient care and allow targeted patient education regarding warning symptoms such as fever, lower abdominal pain, or leg swelling, which may indicate lymphocele-related infection or thromboembolic events. However, such approaches warrant prospective evaluation. To the best of our knowledge, this is the first evidence supporting aLC at discharge as an early risk factor for subsequent sLC after RARP.

Next to aLC at the time of discharge, further significant predicting risk factors for development of sLC are the body-mass-index (BMI), the number of resected lymph nodes and longer operative time [16–18]. The associations of BMI and the number of resected lymph nodes with the rate of sLC were also observed in cohorts undergoing surgery for gynecological malignancies [19] and colorectal cancer [20]. Our analyses showed that patients with obesity (BMI ≥ 30) not only had a higher risk of developing LCs but also derived significantly less benefit from PF than non-obese patients (BMI < 30). A plausible explanation is reduced pelvic working space and greater perivesical fat in obesity, limiting the flap’s ability to keep the peritoneal cavity open and thereby favoring loculation of lymphatic fluid. Beyond local anatomical factors, obesity is also associated with an increased thromboembolic risk such as deep venous thrombosis [21, 22] and consequently a higher likelihood of perioperative anticoagulation [23]. While some studies have reported an association between the treatment with low molecular weight heparin and higher lymphocele incidence [24, 25], the available evidence remains inconsistent [26]. Taken together, elevated BMI may contribute to lymphocele development through a multifactorial interplay, including anatomical constraints within the pelvis as well as impaired lymphatic sealing, which may result from obesity-related compromised wound healing and more intensive perioperative anticoagulation due to increased thromboembolic risk.

Whereas BMI is difficult to modify, this underscores the importance of carefully assessing not only the indication for pelvic lymph node dissection but also its extent. Existing evidence suggests that LCs are more common in extended compared to limited PLND [17]. Recent studies further indicate that extended pelvic lymph node dissection (ePLND) may reduce metastasis rates compared with a limited template, while biochemical recurrence outcomes appear comparable [4]. This has renewed the discussion about the potential oncologic value of ePLND, particularly in intermediate- and high-risk prostate cancer. To date, earlier evidence had not demonstrated a clear oncologic benefit of PLND, and the procedure carries an inherent risk of postoperative morbidity. A population-based study from 2023 reported no improvement in oncological outcomes, including survival, among patients who underwent PLND [27]. However, this analysis was limited to patients with low-risk prostate cancer. In addition, a randomized trial in intermediate- and high-risk patients found no advantage of extended over limited PLND with respect to early oncologic outcomes [28]. Since clear evidence had been lacking, the EAU guideline on prostate cancer recommends a risk-adapted approach regarding lymph-node involvement in intermediate and high-risk groups [29]. To better assess the individual risk of lymph node metastases, several risk calculators like the Briganti or MSKCC nomograms have been developed [30, 31]. While these tools show high sensitivity, this is offset by a relatively low specificity [32]. If PLND is performed, the EAU recommends an extended PLND template [29].

These considerations are directly relevant for the interpretation of the present MA, as heterogeneity across the six included RCTs concerned pelvic lymph node dissection among other surgical aspects. Both the extent of PLND (limited vs. extended) and the applied dissection templates varied between studies and centers. As the extent of lymphatic dissection directly influences lymphatic output and lymphocele volume, such variability may modulate the protective capacity of PF. To address this, subgroup analyses stratified by lymph node yield (< 15 vs. ≥ 15 resected nodes) were performed and demonstrated no evidence of effect heterogeneity, indicating a consistent protective effect of PF across different extents of lymph node dissection. Although randomization within individual trials mitigates systematic bias and subgroup analyses showed consistent effects across different lymph node yields, residual inter-study variability in PLND extent and templates cannot be entirely excluded and should be considered when interpreting pooled results.

Beyond differences in PLND, the included trials also varied with respect to PF construction and fixation techniques. In the PIANFORTE study [5] and the PLUS trial [10], a two-point fixation was used as first introduced by Lebeis et al. [33]. The PerFix study [8] used a running suture to the pubic bone, whereas the ProLy study [7] used a four-point fixation along the arcus tendineus fasciae pelvis and the PELYCAN [6] used a four-point fixation right and left of the anastomosis at the pelvic floor. The Michl-technique used ventral fixation to the plexus Santorini and to the right and left lateral endopelvic fascia [9]. However, all interventions used a similar principle: creating an opening between the pelvis and the peritoneal cavity to allow lymphatic fluid to flow back into the intraperitoneal space for absorption. Nevertheless, heterogeneity in flap construction and fixation may influence the effectiveness of lymphatic shunting and limit surgical standardization and reproducibility across studies; although the effect was consistently favorable across trials, this should be considered when interpreting pooled results. In a recent publication, Gamal et al. [34] described another PF technique: the so-called “bunching” technique was evaluated in 2267 patients, which were propensity score matched in a 3:1 ratio. The PF group showed 0.0% sLCs with a minimum follow-up (FU) of 3 months. FU was extended for a year if aLC was present within the first 3 months of FU. While these findings are encouraging, longer FU and prospective randomized evaluation is required. Taken together, the growing number of technically distinct PF variants highlights the current lack of a standardized best-practice approach. Future comparative studies directly evaluating different flap construction and fixation techniques are warranted to identify the most effective and reproducible strategy and to facilitate consistent clinical implementation.

Another difference between studies is the usage of intraoperative drains. While one study used drains in all patients [5], some applied the use facultatively [7, 9] and others did not use drains at all [6, 8] or did not comment on drainage use [10]. Since there is evidence from meta-analyses that postoperative drainage in RARP does not affect the perioperative course and has no effect on sLC this inhomogeneity seems negligible [35, 36].

Remaining differences are the individual surgical factors (i.e. different surgeons), for which only the PELYCAN study used stratified randomization [6]. Furthermore, the usage of clips vs. cautery vs. vessel sealing during PLND was handled differently: The PerFix study did not allow clips, whereas in the PELYCAN, ProLy or Michl-technique surgeons were free to choose [6–9]. The PIANOFORTE and PLUS trial did not comment on this topic [5, 10]. This difference, too, seems negligible since the use of either clipping or coagulation could not have been linked to LC formation in a previous RCT [37], suggesting that these technical variations are unlikely to have confounded the present results.

Considering the overall complication profile, PF placement appears to be a safe surgical technique. Both the classical and the IPD meta-analyses demonstrated a significant reduction in overall complications among patients receiving PF, most likely driven by fewer lymphoceles and the associated complications (e.g., infections, thrombotic events), along with a reduced need for subsequent interventions such as drainage or antibiotic therapy. When differentiating by severity, this effect was mainly observed for minor complications, while major complication rates were comparable between groups. Additionally, from a surgical point of view, PF placement is considered safe, as it requires only a few simple steps that do not involve risk-prone maneuvers.

Although every additional surgical step may prompt concerns about operating time, PF placement requires only a few minutes and did not result in relevant time delays in the majority of trials. In PerFix, both groups had identical operative times, and in PLUS the difference (± 1.5 min) was minimal [8, 10]. Also for the Michl-technique, no differences in overall surgical time were reported [9]. PIANOFORTE and ProLy even reported shorter operative times in the PF groups (12 and 9 min, respectively), though without statistical significance [5, 7]. Only the PELYCAN trial showed a modest yet statistically significant increase of 11 min [6]. Considering the clinically meaningful reduction in lymphocele-related morbidity, the modest additional operative time required for PF placement represents a favorable balance between procedural effort and clinical benefit.

From such a cost–benefit perspective, PFs should be compared with a range of other prophylactic strategies that have been proposed for lymphocele prevention after PLND. Approaches aimed at sealing lymphatic vessels, including ligation, clipping, or mono- and bipolar coagulation, have been evaluated in comparative settings; however, available data have not demonstrated a consistent and reproducible reduction in clinically relevant sLCs [37–39]. Material-based strategies such as fibrin glue [40], hemostatic patches [41, 42] or vessel-sealing devices have also been explored [43]. While some studies reported potential effects on postoperative fluid collections or lymphocele size [41], these approaches failed to show consistent reductions in sLCs [42, 43] and are associated with additional costs and the use of foreign material. Internal or peritoneal fenestration techniques, including the P.L.E.A.T. approach, represent conceptually related surgical strategies to facilitate lymphatic drainage, based on a comparable physiological rationale to PF placement [44, 45]. However, the evidence base supporting fenestration techniques is considerably less robust than that available for PF [11, 14, 46]. Taken together, compared with alternative prophylactic strategies, PFs combine effective physiological shunting supported by strong evidence with surgical simplicity and the absence of additional materials or devices.

However, these advantages should be interpreted within the surgical context of the existing evidence. The evidence synthesized in the present IPD-MA is derived from RCTs conducted in the setting of transperitoneal RARP with PLND, and the observed reduction in lymphocele-related morbidity should primarily be interpreted in this context.

For alternative operative approaches, including extraperitoneal RARP and Retzius-sparing techniques, the role of PF is less well defined. The current literature addressing PF concepts in these settings is limited and largely indirect, with available data being predominantly retrospective [47, 48] and therefore not allowing firm conclusions regarding efficacy. While the underlying physiological rationale may appear plausible, the transferability of results obtained in transperitoneal RARP to alternative surgical approaches cannot be assumed and remains insufficiently supported by high-level evidence.

### Limitations

This MA is limited by the lack of IPD of one of the six RCTs. Furthermore, the difference in length of FU (ranging from median FU between 90 and 1128 days) and different FU protocols (in-person FU including ultrasonography vs. questionnaire-based FU) could have biased the results.

In addition, the evidence underlying this IPD-MA is derived from RCTs in the setting of transperitoneal RARP with PLND. Consequently, the generalizability of our findings to alternative operative approaches, such as extraperitoneal or Retzius-sparing prostatectomy, is limited.

### Strengths

This MA is the first one, which combines data of all six RCTs on PF for prophylaxis of LC. Furthermore, it is the only one that uses IPD, which was available for five of the six RCTs.

## Conclusion

Creation of PF after RARP with PLND is a safe, simple and inexpensive technique that significantly reduces sLCs requiring intervention. Once established, PF placement does not significantly prolong operative time. Next to BMI and the number of removed lymph nodes, aLCs at the time of discharge represent a significant risk factor for the subsequent occurrence of sLCs. This subgroup should therefore be monitored more closely. The evidence generated by this meta-analysis supports consideration of targeted postoperative screening strategies of patients undergoing RARP with PLND and PF placement as the new standard of care to reduce LC-related morbidity.”

## Supplementary Information

Below is the link to the electronic supplementary material.

Supplementary Material 1

## Data Availability

The datasets generated and/or analyzed during the current study are available from the corresponding author on reasonable request.
