# Supplementary material for: Peritoneal flaps for the prevention of lymphoceles after robot-assisted radical prostatectomy-a systematic review and IPD-meta-analysis of randomized controlled trials
Source: World J Urol. 2026 Feb 24;44(1):193. doi: 10.1007/s00345-026-06254-y (PMC12932341; doi:10.1007/s00345-026-06254-y)
Supplement: Supplementary file 1 — Supplementary Material 1 [file 345_2026_6254_MOESM1_ESM.docx]

# Supplementary Materials

**Peritoneal flaps for the prevention of lymphoceles after robot-assisted radical prostatectomy**

A Systematic Review and IPD-Meta-Analysis of randomized controlled trials

Gloria Baumann^2,1,3*^, Caelán Max Haney-Aubert^1,2,3^ ^*^, Victoria Luise Simone Wieland^1^, Jiri Lehmberg^3^, Wahid Fattal^3^, Christian Gilfrich^4^, Maximilian Burger^5^, Vladimir Student Jr.^6^, Joseph Wagner^7^, Simon Gloger^8^, Maurice Stephan Michel^1^, Karl-Friedrich Kowalewski^1,2,3^, Manuel Neuberger^1^

^1^ Department of Urology and Urosurgery, University Medical Center Mannheim, Medical Faculty Mannheim at Heidelberg University, Germany

^2^ German Cancer Research Center (DKFZ) Heidelberg, Division of Intelligent Systems and Robotics in Urology (ISRU), Heidelberg Germany

^3^ DKFZ Hector Cancer Institute at the University Medical Center Mannheim, Mannheim, Germany

^4^ Department of Uroloogy, St. Elisabeth Hospital Straubing, Straubing, Germany

^5^ Department of Urology, University of Regensburg, Caritas St. Josef Medical Center, Regensburg, Germany

^6^ Department of Urology, University Hospital Olomouc, Olomouc, Czech Republic

^7^ Department of Urology, Hartford Healthcare Medical Group, Hartford Hospital, Hartford, CT, USA

^8^ Center for Minimally Invasive and Robotic Urology, Augusta Hospital Bochum, Witten/Herdecke University, Bochum, Germany

* These authors contributed equally as first authors.

Correspondence to:

Dr. med. Caelán Max Haney-Aubert

Department of Urology and Urologic Surgery, University Medical Centre Mannheim (UMM)

Medical Faculty Mannheim

Heidelberg University

Theodor-Kutzer-Ufer 1-3

68167 Mannheim, Germany

Tel. +49 621 383 2201

Fax: +49 621 383-1471

Email: [caelan.haney@dkfz-heidelberg.de](mailto:karl-friedrich.kowalewski@umm.de)

Number of Supplementary Tables: 3

Number of Supplementary Figures: 1

# Supplementary Tables

| Trial | Design | Country | Registration | Recruitment Period | Primary Outcome | Randomized  (Flap/No FLAP) | IPD |
| --- | --- | --- | --- | --- | --- | --- | --- |
| PROLY  (2022) | Multicenter, randomized,  double-blind | Germany | DRKS00015720 | Nov 2018 –  Aug 2020 | Total lymphocele rate | 262 / 268 | Available |
| PERFIX  (2023) | Single-center, randomized,  single-blind | Czech Republic | NCT04853095 | Dec 2019 –  Jun 2021 | Symptomatic lymphocele rates | 130 / 130 | Available |
| PIANOFORTE (2020) | Multicenter, randomized,  single-blind | Germany | DRKS00011115 | Mar 2017 –  Dec 2017 | Symptomatic lymphocele rates at discharge/90d | 108 / 124 | Available |
| PLUS  (2023) | Single-surgeon, randomized,  assessor-blinded | USA | NCT03567525 | Sep 2018 –  May 2021 | Symptomatic and asymptomatic lymphoceles | 113 / 112 | Available |
| PELYCAN (2023) | Single-center,  phase 3 randomized,  double-blind | Germany | DRKS00016794 | Sep 2019 –  Dec 2021 | Symptomatic lymphocele requiring intervention | 277 / 274 | Available |
| Pose Trial (2025) | Single-center | Germany | NCT06284135 | Jun 2017 –  Oct 2019 | Lymphoceles requiring intervention | 531 / 549 | Not Available |

**Table S1: Overview of Included Studies on Peritoneal Flaps in RARP** (IPD = individual patient data)

| TRIAL | INTERVENTION DESCRIPTION | FOLLOW-UP | BLINDING | DESCRIPTION OF LYMPHADENECTOMY |
| --- | --- | --- | --- | --- |
| PROLY (2022) | Four-point PF fixation along the arcus tendineus fasciae pelvis | Physical examination and US at discharge, 30 day- and 90-day follow-up | Patient blinding, outcome assessor blinding, intraoperative randomization | Standard-template ePLND with deep bilateral incisions along the lateral peritoneal groove down to the common iliac vessels; lymphatics sealed with electrocautery and clips |
| PERFIX (2023) | PF fixation to pubic bone using a running suture (PerFix) | US at catheter extraction, low-dose CT of pelvis after 6 weeks, FU every 3 months, additional low-dose CT of pelvis at 6-month intervals if positive | Patient blinding, staff blinding, surgeon informed on day of operation, unclear if assessor blinding, unclear when unblinding took place | Bilateral ePLND including obturator, external and internal iliac nodes with the ureter as cranial boundary; lymphatics sealed using bipolar coagulation only |
| PIANOFORTE (2020) | Two-point PF fixation to lateral/anterior bladder wall (Lebeis technique) | Clinical assessment at discharge and at 90 days, long-term follow-up (median of 43 months) | Outcome assessor blinding, patient blinding, randomization by operating surgeon (timing not specified) | Standardized bilateral PLND via transperitoneal approach; electrocautery and clips permitted; pelvic drain placed in all patients |
| PLUS (2023) | Two-point PF fixation to lateral/anterior bladder wall (Lebeis technique) | US at approximately 3 months | Blinded outcome assessment, intraoperative randomization | Extended pelvic lymph node dissection (ePLND) performed in all patients |
| PELYCAN (2023) | Four-point PF fixation at the pelvic floor (right and left of the anastomosis) | US at discharge and written survey at 6 months | Patient blinding and outcome assessors blinding up to complete FU, intraoperative randomization | Risk-adapted PLND: non-extended PLND for low/intermediate risk; ePLND for high-risk per EAU guidelines (external iliac, obturator, internal iliac regions) |
| POSE TRIAL (2025) | PF fixation ventrally to Santorini plexus and bilaterally to endopelvic fascia (Michl technique) | Not specified | No blinding reported | Bilateral pelvic lymph node dissection; extent not further specified |

**Table S2: Description of PF Techniques, Follow-Up, Blinding and Lymphadenectomy** (ePLND = extended pelvic lymphadenectomy; FU = follow-up; PF = peritoneal flap; US = ultrasound)

| Predictor | OR | 2.5% CI | 97.5% CI | Significance (P Value) |
| --- | --- | --- | --- | --- |
| (Intercept) | 0.00 | 0.00 | 0.05 | 0.00* |
| Age | 1.02 | 0.99 | 1.05 | 0.24 |
| BMI | 1.06 | 1.01 | 1.11 | 0.01* |
| PSA | 1.00 | NA | 1.00 | 0.82 |
| Lymph node count | 1.01 | 0.98 | 1.04 | 0.43 |
| Lymphoceles at discharge | 3.38 | 2.08 | 5.38 | 0.00* |
| Peritoneal flap | 0.39 | 0.24 | 0.62 | 0.00* |

**Table S3: Predictors of Symptomatic Lymphoceles requiring Intervention: fixed-effects estimates of the multivariable mixed-effects logistic regression model** (BMI: Body mass index; CI: confidence interval; OR: odds ratio; PSA: prostate specific antigen; * statistically significant at p < 0.05)

# Supplementary Figures


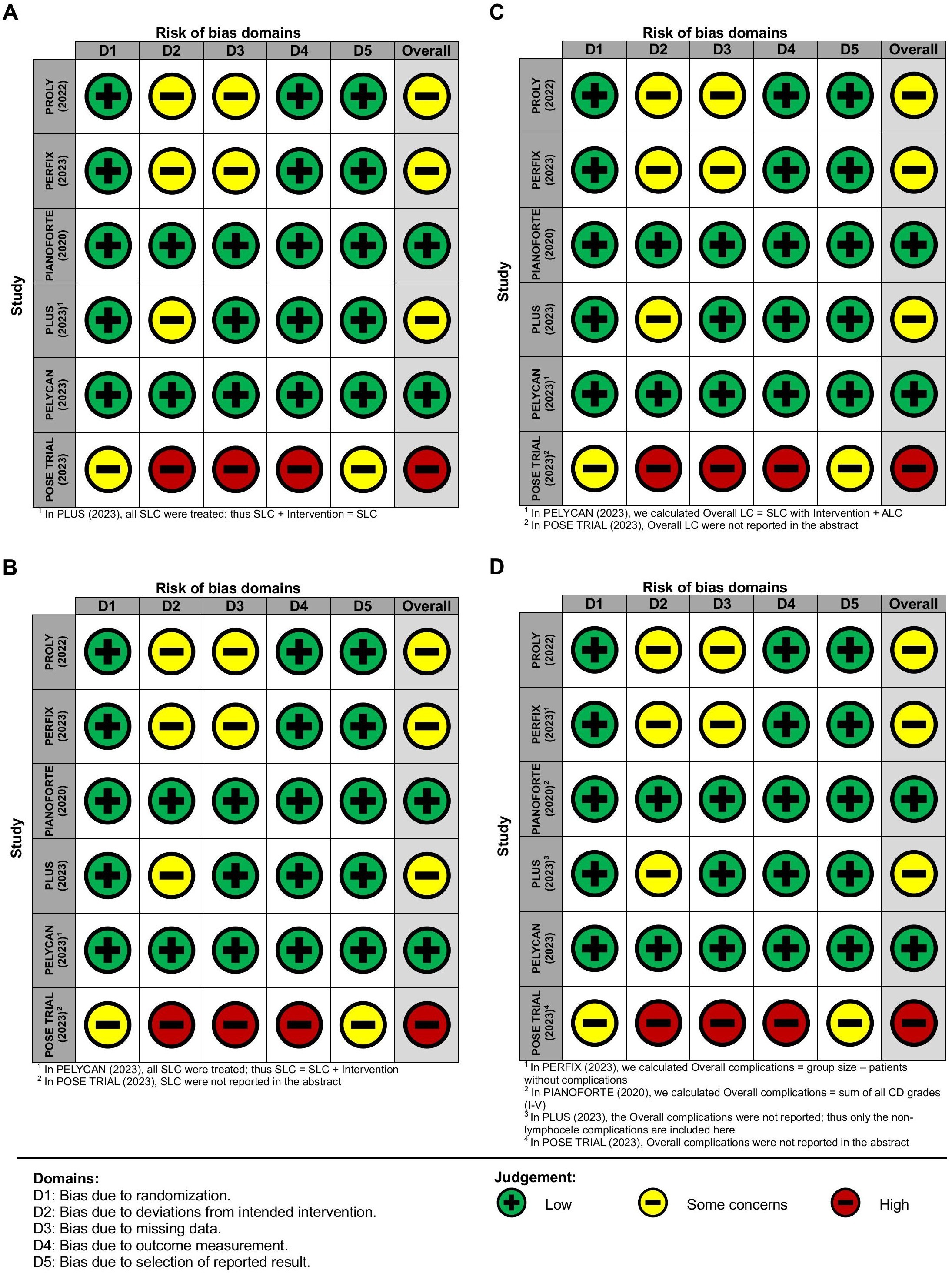


**Figure S1: Risk of Bias Assessment: Panel A:** SLCs requiring intervention; **Panel B:** SLCs; **Panel C:** Overall LCs; **Panel D:** Overall complications. The traffic-light plot shows domain-level and overall RoB 2 judgments per trial. Judgments: green = low risk of bias; yellow = some concerns; red = high risk of bias
